# Effect of pomegranate seed oil on egg production, egg quality and yolk fatty acid deposition in laying hen

**DOI:** 10.1002/vms3.1296

**Published:** 2023-10-17

**Authors:** Alireza Gharagozloo, Farshid Kheiri, Javad Nasr, Mostafa Faghani

**Affiliations:** ^1^ Department of Animal Science Shahrekord Branch Islamic Azad University Shahrekord Iran; ^2^ Department of Animal Science Faculty of Agriculture Saveh Branch Islamic Azad University Saveh Iran

**Keywords:** blood lipids, conjugated fatty acid, laying hen, plant seed oil, production rate

## Abstract

**Background:**

Pomegranate seed oil (PSO) contains punicic acid as well as conjugated linolenic acid isomers, including α‐eleostearic and catalpic acids, along with phytosterols, especially β‐sitosterol, campesterol and stigmasterol, with lipotropic impact and egg fortifying effect in laying hens.

**Objectives:**

The present experiment was designed to examine the effects of PSO on egg production, egg quality, blood lipids and yolk fatty acid deposition in laying hens.

**Methods:**

A total of 360 Hy‐line laying hens (w‐80), at 25 weeks of age, were randomly allotted to five dietary treatments in a completely randomized design during a 10‐week period. Experimental treatments consisted of a basal diet or supplementation of 1, 2, 3 and 4 g PSO/kg to basal diet. Performance indicators and fatty acid composition of egg yolk were evaluated during different experimental periods. Blood lipid attributes were evaluated at the end of the experiment.

**Results:**

Dietary supplementation of 4 g PSO/kg feed increased daily feed intake and egg production rate of laying hens (*p* < 0.05). There was not any significant influence of experimental treatments on egg quality, whereas an increasing trend observed in egg yolk colour of hens received dietary graded levels of PSO. Dietary supplemental 4 g/kg PSO increased the proportion of yolk poly‐unsaturated fatty acid (PUFA) concentration (*p* < 0.05). Furthermore, the PUFA to saturated fatty acid ratio increased after dietary supplementation of 1 or 4 g/kg PSO (*p* < 0.05). Serum concentration of cholesterol, triacylglycerol and low‐density lipoprotein decreased in response to the supplementation of more than 3 g PSO/kg in the feed (*p* < 0.05).

**Conclusions:**

In conclusion, dietary supplementation with 4 g/kg PSO improved production rate and decreased blood lipids in laying hens. Moreover, dietary supplemental PSO modified yolk fatty acid deposition without detrimental effects on the egg quality.

## Graphical abstract

Dietary supplementation of 4 g/kg Pomegranate seed oil (PSO) increased daily feed intake, egg production rate, and proportion of yolk poly‐unsaturated fatty acid (PUFA) concentration. The PUFA to saturated fatty acid ratio increased after dietary supplementation of 1 g/kg or 4g/kg PSO. In conclusion, using 4 g/kg PSO in the feed improved production rate and decreased blood lipids in laying hens. Also, PSO modified yolk fatty acid deposition without detrimental effects on the egg quality.

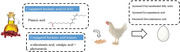

## 1 INTRODUCTION

During recent years, the positive effects of bioactive components on human health have gained growing attention. As a result, different nutritional strategies are applied to enrich human food with these compounds. In this regard, the modification of animal nutrition to manipulate fat composition of derived products is a common process. Egg is one of the cheapest animal products and has potential to provide certain nutrients for consumers (Surai & Sparks, 2001). Moreover, the possibility of using table eggs as a functional food to transfer conjugated fatty acid (CFA) to the egg yolk has already been verified (Hur et al., 2003). The CFAs are isomers of poly‐unsaturated fatty acids (PUFA) with conjugated double bonds and are considered effective in prevention and treatment of chronic diseases. Furthermore, CFA has another conjugated form named conjugated linoleic acid (CLA) which shares beneficial physiologic properties against atherosclerosis, obesity, tumour and hypertension (Lee et al., 1994). However, incorporating CFA to the egg yolk might deteriorate sensory properties and shelf life of the egg (Dunn‐Horrocks et al., 2011). Moreover, some parameters of the egg quality were compromised in response to the supplementation of hen diet with CLA oil, whereas quality was not affected when CLA was combined simultaneously with some other fatty acids (Kim et al., 2007). In this respect, plant seeds such as flaxseed or pomegranate seed oil (PSO) are commonly added to the diet of laying hen in order to fortify egg with alpha‐linolenic acid and its long‐chain anabolic derivative, which have shown health benefits in terms of inflammatory processes (Calder, 2017). Conjugated linolenic acid (CLnA) contains three conjugated double bonds as well as the carbon structure (18:3) of a‐linolenic acid. Punicic acid (9cis, 11trans, 13cis‐cLNA; 9c, 11t, 13c‐CLNA) is a type of CFA contained in PSO at a level of about 72% (Suzuki et al., 2001). The PSO also contained cLNA isomers other than punicic acid, including α‐eleostearic and catalpic acids, along with phytosterols, especially β‐sitosterol, campesterol and stigmasterol, which are also considered to induce health beneficial effects. The PSO was reported to decrease hepatic triacylglycerol accumulation in obese hyperlipidemic rats (Arao et al., 2004) and decreased plasma cholesterol and low‐density lipoprotein (LDL) concentration in rats fed with an atherogenic diet (Elbandy & Ashoush, 2012). On the other hand, improvement in egg production was reported when laying hen received diets supplemented with 2.5% PSO (Kostogrys et al., 2017). Moreover, successful transfer of CLA to the egg yolk was observed when layers received diets supplemented with PSO (Kostogrys et al., 2017) or mix of flaxseed and PSO (Ngo Njembe et al., 2021). Although research using CLA as a functional food is found in the literature, the effect of seed oils containing CLA isomers such as dietary punicic acid on egg deposition of laying hen is limited. Moreover, the effect of PSO on egg quality and blood lipids is less investigated. Thus, the present experiment was designed to investigate effects of dietary supplementation with 1, 2, 3 and 4 g PSO/kg on egg production variables, egg qualitative indices, serum biochemical attributes and yolk fatty acid deposition in laying hens.

## 2 MATERIALS AND METHODS

All animal work in the present study was conducted according to the comprehensive guidelines of the animal welfare adopted by Federation of Animal Science Societies (FASS) (2010). The experiment was conducted according to the regulations and guidelines established by this committee.

### 2.1 Preparation of pomegranate seed and pomegranate seed oil

The pomegranate was purchased from a local market as fresh Iranian pomegranate (Malas Saveh). The seeds were manually removed from arils and then air dried under ambient condition and finely ground in a grinder to pass 40–33 mesh. The seed powder was finally packed and stored at −18°C. In order to prepare the PSO, pomegranate seeds were oiled by cold pressing method and applied in dietary treatments.

### 2.2 Active components and fatty acid composition of pomegranate seed oil

The fatty acid composition of PSO (Table 1) was determined by a direct method (O'Fallon et al., 2007) for fatty acid methyl ester synthesis using a gas chromatograph (YOUNG LIN ACME). Determination of total antioxidant activity applied through the methods described in the work of Madaan et al. (2011) and total phenolic contents (Table 2) assayed according to the method of Dewanto et al. (2002).

**TABLE 1:** Fatty acid composition of pomegranate seed oil.

	Quantity
C14:0 (%)	0.03
C16:0 (%)	3.55
C16:1 (%)	0.05
C18:0 (%)	2.58
C18:1 (%)	6.75
C18:2 (%)	7.58
C20:3 (%)	0.51
C20:4 (%)	0.78
C18:3 (%)	78.18

**TABLE 2:** Antioxidant activity and total phenolics of pomegranate seed oil.

	Quantity
Antioxidant activity (%)	7.55
Total phenolics (mg GAE/g)	10.52

### 2.3 Birds, diets and experiment design

Three hundred and sixty Hy‐line‐laying hens (w‐80), at 25 weeks of age, were weighed individually and then randomly allocated into 5 groups (60 birds) and 6 replicates. Each replicate contained 12 birds housed in individual 4‐level cages (120 cm × 60 cm × 50 cm) under the same management and environmental conditions. Experimental treatments consisted of five groups of birds fed a basal diet (CTL) or basal diet supplemented with either 1, 2, 3 or 4 g PSO/kg during a period of 10 weeks. Iso‐energetic and iso‐nitrogenous experimental mash diets were formulated to be used in this experiment. Diet formulation was performed according to Hy‐Line W‐80 breeder recommendation guideline and according to their production phase (Table 3). Feed and water were ad libitum provided. The birds were exposed to continuous daily lighting for 17 h. Before the beginning of the trial, a 2‐week pre‐experiment period was carried out to ensure the eggs production rate was similar among the experimental groups.

**TABLE 3:** Dietary composition and nutrients.

	Experimental diet^a^				
Items	CTL	PSO1	PSO2	PSO3	PSO4
Ingredients (g/kg)					
Corn (80 g/kg crude protein)	599.95	600.65	601.55	602.65	603.75
SBM (440 g/kg crude protein)	214	214.40	214.40	214.40	214.4
Wheat bran (155)	36	36	36	36	36
Pomegranate seed oil	0	1	2	3	4
Soybean oil	16.70	14.60	12.60	10.60	8.50
Dicalcium phosphate	21.10	21.10	21.10	21.10	21.10
Calcium carbonate	98.30	98.30	98.30	98.30	98.30
dl‐Methionine	2.60	2.60	2.60	2.60	2.60
l‐Lysine	1.50	1.50	1.50	1.50	1.50
Choline	0.25	0.25	0.25	0.25	0.25
Vitamin and mineral premix^b^	5	5	5	5	5
Sodium chloride	1.70	1.70	1.70	1.70	1.70
NaHCo_3_	2.90	2.90	2.90	2.90	2.90
Calculated nutrient level (as fed basis)					
ME (Kcal/kg)	2711	2711	2711	2711	2711
Crude protein (g/kg)	150.5	150.5	150.5	150.5	150.5
Lysine (g/kg)	8.4	8.4	8.4	8.4	8.4
Met + Cys (g/kg)	7.4	7.4	7.4	7.4	7.4
Crude fibre (g/kg)	30	30	30	30	30
Linoleic acid (g/kg)	21.5	20.79	20.03	19.27	18.51
Sodium (g/kg)	1.7	1.7	1.7	1.7	1.7
Chloride (g/kg)	1.7	1.7	1.7	1.7	1.7
Calcium (g/kg)	42.7	42.7	42.7	42.7	42.7
Available phosphorous (g/kg)	4.7	4.7	4.7	4.7	4.7
Analysed values (as fed basis)					
Crude protein (g/kg)	149.3	149.2	149.3	149.5	149.5
Calcium (g/kg)	42.84	42.75	42.73	42.84	42.75
Total phosphorous (g/kg)	7.01	7.05	7.01	7.01	7.01

Abbreviation: SBM, soybean meal.

^a^ CTL: a basal diet; PSO1, PSO2, PSO3 and PSO4 represent a basal diet supplemented with 1, 2, 3 and 4 g/kg pomegranate seed oil, respectively.

^b^ Mineral supplement per kg of diet: 2.4 mg copper, 0.34 Mg iodine, 30 mg iron, 29.76 mg manganese, 0.008 mg selenium, 25.87 mg zinc. Vitamin supplement per kg of diet: 3520 IU vitamin A, 0.59 mg vitamin B1, 1.6 mg vitamin B2, 13.86 mg niacin, 3.13 mg pantothenic acid, 1 mg vitamin B6, 0.06 mg biotin, 80 mg choline, 0.004 mg vitamin B12, 19.9 mg vitamin B9, 1000 IU vitamin D3, 8.8 IU vitamin E, 0.88 mg vitamin K.

### 2.4 Performance indicators and egg quality

Daily feed intake (DFI), egg production rate and egg weight were recorded for each replicate. Furthermore, egg mass (g egg/hen/day) and feed conversion ratio (g feed consumed/g egg mass) were also calculated. Collecting and weighting of eggs were carried out at the same time every afternoon.

A total of four fresh laid eggs/cages were collected from each treatment on the same day. After weighing the individual eggs, a force reader (Orka Food Technology) was used to break each egg to measure eggshell breaking strength. Eggshell thickness is presented as an average value based on the thickness of eggshell measured at three different places (upper end, lower end and the middle) using a micrometer screw gauge (Suce Measuring Instrument Co., Ltd.). An automatic egg quality analysis instrument was used to measure the haugh units and yolk colour (EMT‐5200, Robotmation Co., Ltd.). The albumen was removed from shells, and shells plus membranes were weighed after 24 h of air drying. Specific gravity of the egg was measured by dipping the eggs in a predetermined salt solutions specific gravity ranging from 1.060 to 1.100 (Peebles & McDaniel, 2004).

### 2.5 Serum biochemical parameters

At the end of the experimental period, blood samples were collected from two birds of each cage and put in non‐heparinized tubes by brachial vein puncture. Serum was separated via 2000 × *g* centrifuge of blood samples for 15 min (SIGMA 4‐15 Lab Centrifuge). Individual serum samples were analysed for total cholesterol, triacylglycerol, high‐density lipoprotein (HDL), LDL, calcium, phosphorus, iron and magnesium with a spectrophotometer using the kit package (Pars Azmoon Co.) according to manufacturer's guidelines.

### 2.6 Fatty acid composition of egg yolk

Total egg yolk lipids were extracted using the FAT Extractor TFE 2000, with liquid carbon dioxide as a solvent (Domagała et al., 2010). After the extraction of fat (described above), the lipids were esterified according to the method described by de Man (1964). A volume of 0.1 mL of extracted fat was placed in the glass test tube of 2 mL capacity, and 0.5 mL of 0.025 M solution of sodium methylate was added. The mixture was heated in a closed tube at 60°C until the mixture was clear. The analysis of fatty acids was carried out using gas chromatograph Trace GC Ultra (Thermo Electron Corporation) with a Supelcowax 10 column (dimensions 30 m × 0.25 mm × 0.25 mm). Helium as a gaseous phase was applied with the flow rate of 5 mL/min. The feeder had the temperature of 220°C. The temperature of the column was kept for 3 min at 60°C and then increased at a rate of 7°C/min up to 200°C and held at this temperature for 20 min. The detector had the temperature of 250°C, and the split flow was 10 mL/min. Peak identification was possible with the aid of an external standard (FIM/FAME Supelco). Fatty acid methyl esters were identified by comparing their retention times with authentic standards (Sigma‐Aldrich) and the Punicic Acid Standard (Larodan Fine Chemicals AB). The average laboratory sample was prepared from 20 randomly selected eggs. The analyses were performed in triplicates.

### 2.7 Statistical analysis

Data for recorded traits were subjected to the analysis of variance procedures appropriate for a completely randomized design using the General Linear Model procedure of SAS 9.2 (SAS Institute Inc.). For all statistical analyses, significance was declared at *p* ≤ 0.05, unless otherwise stated. The Tukey test was used for multiple treatment comparisons. Results are presented as least square means ± SEM.

## 3 RESULTS

### 3.1 Performance indicators and egg quality

Effects of experimental treatments on egg production and egg qualitative variables are shown in Tables 4 and 5, respectively. Increased DFI and egg production rate were observed as a response to 4 g dietary supplementation of PSO/kg compared to CTL diet (*p* < 0.05). Moreover, birds received 4 g/kg PSO in their feed displayed greater DFI and egg production than those fed 1 g/kg PSO supplemented diets (*p* < 0.05). Egg quality was not affected by experimental treatments; however, an increasing trend observed in egg yolk colour of hens received graded levels of PSO by 7.4% compared to control group.

**TABLE 4:** Egg production variables of laying hens during 25–35 weeks age.

	Experimental treatments^*^					SEM	*p*‐Value
Parameters	CTL	PSO1	PSO2	PSO3	PSO4		
Feed intake (g/d)	90.5^b^	92.2^b^	96.0^a,b^	94.9^a,b^	100.2^a^	3.75	0.011
Egg mass (g)	57.5	59.1	57.8	57.3	57.3	3.06	0.675
FCR (g/g)	1.57	1.56	1.66	1.65	1.74	0.14	0.522
Egg weight (g)	60.5	61.8	59.7	59.4	58.5	2.71	0.411
Egg production (%)	95.1^b^	95.6^b^	96.8^a,b^	96.4^a,b^	98.0^a^	1.95	0.031

*Note*: Means in the same row with different superscripts (a and b) differ significantly (*p* < 0.05).

Abbreviation: FCR, feed conversion ratio.

* CTL: a basal diet; PSO1, PSO2, PSO3 and PSO4 represent a basal diet supplemented with 1, 2, 3 and 4 g/kg pomegranate seed oil, respectively.

**TABLE 5:** Egg qualitative traits of laying hens.

	Experimental treatments^*^					SEM	*p*‐Value
Parameters	CTL	PSO1	PSO2	PSO3	PSO4		
Yolk colour	6.75	6.75	7.11	7.25	7.25	0.21	0.063
Hugh unit	86.9	85.4	85.9	86.73	87.51	2.62	0.831
Specific gravity (g/mm)	1.06	1.07	1.07	1.07	1.07	0.002	0.282
Eggshell weight (g)	5.70	5.72	5.73	5.91	5.72	0.32	0.475
Eggshell strength (kg/cm^3^)	3.01	3.01	3.05	3.06	3.06	0.09	0.728
Eggshell thickness (mm)	0.31	0.32	0.31	0.31	0.31	0.01	0.612

*Note*: Means in the same row with different superscripts (a and b) differ significantly (*p* < 0.05).

* CTL: a basal diet; PSO1, PSO2, PSO3 and PSO4 represent a basal diet supplemented with 1, 2, 3 and 4 g/kg pomegranate seed oil, respectively.

### 3.2 Serum biochemical parameters

As indicated in Table 6, the supplementation of PSO to the feed decreased serum triacylglycerol content (*p* < 0.05). Similarly, reduction in blood cholesterol concentration observed with dietary 3 or 4 g PSO/kg supplementation (*p* < 0.05). In comparison to CTL diet, serum HDL content increased in hens received 2–4 g PSO/kg feed (*p* < 0.05), whereas blood LDL concentration decreased with the inclusion of 3 or 4 g PSO/kg feed (*p* < 0.05).

**TABLE 6:** Serum biochemical parameters.

		Experimental treatments^*^				SEM	*p*‐Value
Parameters	CTL	PSO1	PSO2	PSO3	PSO4		
Cholesterol (mg/dL)	210.0^a^	138.7^a,b^	129.2^a,b^	104.2^b^	103.0^b^	45.51	0.007
Triacylglycerol (mg/dL)	645.8^a^	515.2^b^	503.0^b^	482.0^b^	479.2^b^	63.50	0.006
HDL (mg/dL)	41.5^b^	41.3^b^	55.1^a^	54.3^a^	54.1^a^	6.25	0.041
LDL (mg/dL)	49.0^a^	48.2^a^	45.2^a,b^	43.4^b^	42.0^c^	2.35	0.001

*Note*: Means in the same row with different superscripts (a, b and c) differ significantly (*p* < 0.05).

Abbreviations: HDL, high density lipoprotein; LDL, low density lipoprotein.

* CTL: a basal diet; PSO1, PSO2, PSO3 and PSO4 represent a basal diet supplemented with 1, 2, 3 and 4 g/kg pomegranate seed oil, respectively.

### 3.3 Fatty acid composition of egg yolk

Fatty acids’ profiles in the egg yolks are shown in Table 7. Dietary supplemental 4 g PSO/kg increased the proportion of yolk PUFA (*p* < 0.05). Moreover, the PUFA to SFA ratio (PUFA/SFA) increased after dietary supplementation of 1 or 4 g PSO/kg (*p* < 0.05). Additionally, total proportion of PUFA and MUFA was greater in egg yolk of birds received 1 g PSO/kg diet (*p* < 0.05). Eicosapentaenoic acid (EPA; C20:5) concentration increased when greater than 2 g PSO/kg added to the feed (*p* < 0.05). Moreover, dietary supplemental 4 g/kg PSO caused higher yolk EPA content than the other PSO levels (*p* < 0.05). Furthermore, the yolk docosahexaenoic acid (DHA; C22:6) content decreased in response to the dietary supplementation of more than 3 g PSO/kg feed compared to CTL and lower levels of supplemental PSO (*p* < 0.05).

**TABLE 7:** Fatty acid profile of egg yolks.

	Experimental treatments^*^					SEM	*p*‐Value
Parameters	CTL	PSO1	PSO2	PSO3	PSO4		
C14:0 (%)	0.82	0.32	0.35	0.35	0.36	0.41	0.722
C16:0 (%)	28.2^a^	25.9^b^	27.17^a,b^	26.72^a,b^	26.77^a,b^	0.48	0.025
C16:1 (%)	3.39	2.82	3.04	3.02	3.12	0.52	0.495
C18:0 (%)	7.81^b^	8.61^a,b^	8.42^a,b^	9.21^a^	8.90^a,b^	0.77	0.013
C18:1 (%)	44.34^a,b^	44.03^a,b^	43.46^a,b^	45.75^a^	41.46^b^	0.92	0.006
C18:2 (%)	12.23^a,b^	13.71^a,b^	13.42^a,b^	10.70^b^	14.31^a^	0.82	0.017
C18:3 (%)	0.24^b^	0.38^a^	0.30^a,b^	0.253^b^	0.32^a,b^	0.20	0.023
C20:3 (%)	0.13	0.133	0.12	0.083	0.135	0.03	0.355
C20:4 (%)	0.21^d^	0.66^c^	0.84^c^	1.17^b^	1.49^a^	0.16	<0.001
C20:5 (%)	0.13^c^	0.20^a,b^	0.18^b^	0.18^b^	0.23^a^	0.02	<0.001
C22:4 (%)	1.66	1.52	1.62	1.47	1.58	0.21	0.676
C22:5 (%)	0.39^b^	0.82^a^	0.46^b^	0.41^b^	0.44^b^	0.18	0.015
C22:6 (%)	0.50^a,b^	0.58^a^	0.45^b^	0.38^c^	0.38^c^	0.11	<0.001
∑SFA	36.82	35.01	35.92	36.30	36.03	1.50	0.245
∑MUFA	47.71^a,b^	46.80^a,b^	46.53^a,b^	48.82^a^	44.60^b^	2.4	0.084
∑PUFA	13.82^b^	17.20^a,b^	16.33^a,b^	14.31^b^	18.14^a^	2.24	0.038
PUFA/SFA	0.38^b^	0.58^a^	0.46^a,b^	0.45^a,b^	0.59^a^	0.07	0.032
PUFA + MUFA	61.75^b^	64.04^a^	62.81^a,b^	62.53^a,b^	62.71^a,b^	0.61	0.021
PUFA + MUFA/SFA	1.71	1.84	1.82	1.73	1.70	0.19	0.243
*ω* − 6/*ω* − 3	13.42^a,b^	9.98^b^	13.33^a,b^	12.61^a,b^	15.32^a^	1.24	0.007

*Note*: Means in the same row with different superscripts (a and b) differ significantly (*p* < 0.05).

Abbreviations: MUFA, mono unsaturated fatty acids; PUFA, poly‐unsaturated fatty acids, SFA, saturated fatty acids.

* CTL: a basal diet; PSO1, PSO2, PSO3 and PSO4 represent a basal diet supplemented with 1, 2, 3 and 4 g/kg pomegranate seed oil, respectively.

## 4 DISCUSSION

The PSO was evaluated for fatty acid profile, antioxidant activity (7.55%) and total phenolic (10.52 mg GAE/g) contents. All these values fell within the physiological range similar to the other Iranian pomegranate cultivars as indicated in the work of Derakhshan et al. (2018).

Dietary supplementation of 4 g/kg PSO increased DFI and egg production rate of laying hens, suggesting the growth promotional function of PSO. Similarly, Arao et al. (2004) found an improvement in feed efficiency of obese rats received diets containing 1% PSO. Moreover, Chin et al. (1994) reported that CLA is a dietary factor that improves the growth and feed efficiency of rats. There are limited information on the effect of PSO on the performance of laying hens. In this respect, Kostogrys et al. (2017) showed that the supplementation of dietary 2.5% PSO improved DFI and laying rate of hens. Therefore, the increased rate of laying in response to PSO might be due to the improvement in palatability and consequently enhanced feed intake. Active components of PSO also possess anti‐oxidative properties, protecting body organs from undesirable effects of free radicals. On the contrary, Ngo Njembe et al. (2021) could not find any effect of dietary supplemental 7 wt% PSO mixed with 7.5% flaxseed oil on the laying performance of laying hens. Thus, further investigations are needed to evaluate the effect of PSO on laying performance.

As Kim et al. (2007) showed an improvement of egg quality in layers fed with a dietary combination of CLA and other fatty acids, we expected that using PSO as a source of CLnA and punicic acid might enhance egg quality. However, egg quality was not significantly affected using PSO, but there was a tendency to increase egg yolk colour with the consumption of higher PSO levels. This is in accordance with the work of Kostogrys et al. (2017) who reported a greater egg yolk colour in laying hens fed diets supplemented with 2.5% PSO. It is likely that higher levels of PSO supplementation compared to this trial made significantly darker colour. Reports have shown beneficial impact of feed additives on nutritional value of egg (Ahmad et al., 2012) which shows the opportunity to enrich the human food through dietary modification of poultry feed but the main obstacle is the subsequent reduction in egg quality (Franczyk‐Żarów et al., 2008) and egg sensory properties (Kostogrys et al., 2017). This is in contrast to our data, suggesting that the inclusion of PSO did not induce any adverse effect on Haugh unit, specific gravity and shell thickness. It shows that PSO could keep proper quality of a typical fresh egg without any detrimental impact.

Fatty acid profile of the egg yolk could be affected by fatty acid composition of hen diet (Ahmad et al., 2012). Hence, blood lipid contents could be in part indicators of egg lipid concentrations. Data showed that serum cholesterol, triacylglycerol and LDL contents decreased in response to supplementation of more than 3 g PSO/kg in the feed. Generally, CFA are known as hypolipidaemic agents (Elbandy & Ashoush, 2012) and PSO containing conjugated octadecatrienoic fatty acids that is considered one of the isomers of CLN. The PSO was reported to decrease plasma total cholesterol and LDL cholesterol in rats receiving an atherogenic diet (Elbandy & Ashoush, 2012). Furthermore, a gradual reduction in serum total cholesterol and LDL of birds fed PSO and linseed oil combination observed in the work of Manterys et al. (2016). In contrast, Du and Ahn (2003) indicated that the dietary supplementation of 2% and 3% CLA increased plasma total cholesterol concentration in broiler chickens. On the other side, dietary PSO supplementation did not affect the serum lipid level of Otsuka long‐evans tokushima fatty rats but decreased triacylglycerol accumulation in their liver (Arao et al., 2004). Results on the effect of CFA on lipid metabolism are inconsistent and it seems that isomer configuration and amount of CLN and CLA are determining factors (Mirmiran et al., 2010).

Generally, an arrangement of lipids formed in the liver for yolk synthesis can be affected by diet (Walzem, 1996). Data obtained on the enrichment of eggs with PUFA is in agreement with findings of Kostogrys et al. (2017) and Ngo Njembe et al. (2021) when birds fed on diets supplemented with PSO or a combination of PSO and flaxseed oil, respectively. In this regard, the deposition of DHA or EPA increased in the egg yolk of birds fed PSO supplemented diets. Similar data in animal products observed when concentrations of EPA and DHA increased in the breast muscle of broiler chickens fed diets containing punicic acid (Szymczyk & Szczurek, 2016). The egg yolk SFA content was not affected by the inclusion of PSO in the feed, whereas the PUFA to SFA ratio increased. Due to the fact that *ω* − 6 and *ω* − 3 are essential fatty acids in human diet, maintaining a proper ratio of *ω* − 6 and *ω* − 3 fatty acids not only improves performance but also prevents health risks (Alagawany et al., 2018). In the current experiment, the *ω* − 6 to *ω* − 3 ratio did not change in response to PSO supplementation to the feed, suggesting a lack of its detrimental impact on cardiovascular and neurodegenerative (Avallone et al., 2019) disease.

## 5 CONCLUSION

Dietary supplementation of PSO enhanced DFI, laying performance and tended to increase egg yolk colour of laying hen received diets containing 4 g/kg PSO. Serum cholesterol, triacylglycerol and LDL contents decreased following supplementation of more than 3 g PSO/kg in the feed, suggesting a hypolipidaemic effect of CFA in the PSO. Yolk deposition of EPA increased in birds fed 4 g/kg PSO added diets, whereas DHA decreased with the same supplemental level. Taken together, these data indicate that the dietary supplementation of 4 g/kg PSO could efficiently improve laying performance and beneficially modify egg fatty acid contents without any undesirable effects on the egg quality.

## AUTHOR CONTRIBUTIONS

*Investigation; conceptualization*: Alireza Gharagozloo. *Conceptualization; methodology; project administration*: Farshid Kheiri. *Conceptualization*: Javad Nasr. *Formal analysis; software*: Mostafa Faghani.

## CONFLICT OF INTEREST STATEMENT

Authors declare no conflicts of interest.

## ETHICS STATEMENT

All animal work in the present study was conducted according to the comprehensive guidelines of the animal welfare adopted by FASS (2010). The experiment was conducted according to the regulations and guidelines established by this committee.

## Data Availability

Author elects to not share data

## References

[vms31296-bib-0001] Ahmad, S. , Ahsan‐ul‐Haq , Yousaf, M. , Sabri, M. A. , & Kamran, Z. (2012). Response of laying hens to omega‐3 fatty acids for performance and egg quality. Avian Biology Research, 5, 1–10.

[vms31296-bib-0002] Alagawany, M. , Farag, M. R. , Dhama, K. , & Patra, A. (2018). Nutritional significance and health benefits of designer eggs. World's Poultry Science Journal, 74, 317–330.

[vms31296-bib-0003] Arao, K. , Wang, Y.‐M. , Inoue, N. , Hirata, J. , Cha, J.‐Y. , Nagao, K. , & Yanagita, T. (2004). Dietary effect of pomegranate seed oil rich in 9cis, 11trans, 13cis conjugated linolenic acid on lipid metabolism in obese, hyperlipidemic OLETF rats. Lipids in Health and Disease, 3, 1–7. 15533261 10.1186/1476-511X-3-24 PMC534798

[vms31296-bib-0004] Avallone, R. , Vitale, G. , & Bertolotti, M. (2019). Omega‐3 fatty acids and neurodegenerative diseases: New evidence in clinical trials. International Journal of Molecular Sciences, 20, 4256. 31480294 10.3390/ijms20174256 PMC6747747

[vms31296-bib-0005] Calder, P. C. (2017). Omega‐3 fatty acids and inflammatory processes: From molecules to man. Biochemical Society Transactions, 45, 1105–1115. 28900017 10.1042/BST20160474

[vms31296-bib-0006] Chin, S. F. , Storkson, J. M. , Albright, K. J. , Cook, M. E. , & Pariza, M. W. (1994). Conjugated linoleic acid is a growth factor for rats as shown by enhanced weight gain and improved feed efficiency. The Journal of Nutrition, 124, 2344–2349. 16856314 10.1093/jn/124.12.344

[vms31296-bib-0007] Derakhshan, Z. , Ferrante, M. , Tadi, M. , Ansari, F. , Heydari, A. , Hosseini, M. S. , Conti, G. O. , & Sadrabad, E. K. (2018). Antioxidant activity and total phenolic content of ethanolic extract of pomegranate peels, juice and seeds. Food Chemical Toxicology, 114, 108–111. 29448088 10.1016/j.fct.2018.02.023

[vms31296-bib-0008] Dewanto, V. , Wu, X. , Adom, K. K. , & Liu, R. H. (2002). Thermal processing enhances the nutritional value of tomatoes by increasing total antioxidant activity. Journal of Agricultural and Food Chemistry, 50, 3010–3014. 11982434 10.1021/jf0115589

[vms31296-bib-0009] de Man, J. M. (1964). Determination of fatty acid composition of milk fat by dual column temperature programmed gas liquid chromatography. Journal of Dairy Science, 47, 546–547.

[vms31296-bib-0010] Domagała, J. , Sady, M. , Grega, T. , Pustkowiak, H. , & Florkiewicz, A. (2010). The influence of cheese type and fat extraction method on the content of conjugated linoleic acid. Journal of Food Composition and Analysis, 23, 238–243.

[vms31296-bib-0011] Du, M. , & Ahn, D. U. (2003). Dietary CLA affects lipid metabolism in broiler chicks. Lipids, 38, 505–511. 12880105 10.1007/s11745-003-1091-z

[vms31296-bib-0012] Dunn‐Horrocks, S. , Pichardo‐Fuchs, M. , Lee, J. , Ruiz‐Feria, C. , Creger, C. , Hyatt, D. , Stringfellow, K. , Sanchez, M. , & Farnell, M. (2011). Effect of omega‐3 enriched layer rations on egg quality. International Journal of Poultry Science, 10, 8–11.

[vms31296-bib-0013] Elbandy, M. A. , & Ashoush, I. S. (2012). Phytochemicals in pomegranate seeds and their effect as hypolipidemic agent in hypercholesterolemic rats. World Journal of Dairy & Food Sciences, 7, 85–92.

[vms31296-bib-0014] Federation of Animal Science Societies (FASS) . (2010). Guide for the care and use of agricultural animals in research and teaching . Federation of Animal Science Societies (FASS).

[vms31296-bib-0015] Franczyk‐Żarów, M. , Kostogrys, R. B. , Szymczyk, B. , Jawień, J. , Gajda, M. , Cichocki, T. , Wojnar, L. , Chlopicki, S. , & Pisulewski, P. M. (2008). Functional effects of eggs, naturally enriched with conjugated linoleic acid, on the blood lipid profile, development of atherosclerosis and composition of atherosclerotic plaque in apolipoprotein E and low‐density lipoprotein receptor double‐knockout mice (apoE/LDLR−/−). British Journal of Nutrition, 99, 49–58. 17678565 10.1017/S0007114507793893

[vms31296-bib-0016] Hur, S.‐J. , Kang, G.‐H. , Jeong, J.‐Y. , Yang, H.‐S. , Ha, Y.‐L. , Park, G.‐B. , & Joo, S.‐T. (2003). Effect of dietary conjugated linoleic acid on lipid characteristics of egg yolk. Asian‐Australasian Journal of Animal Sciences, 16, 1165–1170.

[vms31296-bib-0017] Kim, J. H. , Hwangbo, J. , Choi, N. J. , Park, H. G. , Yoon, D. H. , Park, E. W. , Lee, S. H. , Park, B. K. , & Kim, Y. J. (2007). Effect of dietary supplementation with conjugated linoleic acid, with oleic, linoleic, or linolenic acid, on egg quality characteristics and fat accumulation in the egg yolk. Poultry Science, 86, 1180–1186. 10.1093/ps/86.6.1180 17495090

[vms31296-bib-0018] Kostogrys, R. B. , Filipiak‐Florkiewicz, A. , Dereń, K. , Drahun, A. , Czyżyńska‐Cichoń, I. , Cieślik, E. , Szymczyk, B. , & Franczyk‐Żarów, M. (2017). Effect of dietary pomegranate seed oil on laying hen performance and physicochemical properties of eggs. Food Chemistry, 221, 1096–1103. 27979064 10.1016/j.foodchem.2016.11.051

[vms31296-bib-0019] Lee, K. N. , Kritchevsky, D. , & Parizaa, M. W. (1994). Conjugated linoleic acid and atherosclerosis in rabbits. Atherosclerosis, 108, 19–25. 7980704 10.1016/0021-9150(94)90034-5

[vms31296-bib-0020] Madaan, R. , Bansal, G. , Kumar, S. , & Sharma, A. (2011). Estimation of total phenols and flavonoids in extracts of *Actaea spicata* roots and antioxidant activity studies. Indian Journal of Pharmaceutical Sciences, 73, 666. 23112402 10.4103/0250-474X.100242 PMC3480753

[vms31296-bib-1021] Manterys, A. , Franczyk‐Zarow, M. , Czyzynska‐Cichon, I. , Drahun, A. , Kus, E. , Szymczyk, B. , & Kostogrys, R. B. (2016). Haematological parameters, serum lipid profile, liver function and fatty acid profile of broiler chickens fed on diets supplemented with pomegranate seed oil and linseed oil. British Poultry Science, 51, 777–779. 10.1080/00071668.2016.1219977 27636015

[vms31296-bib-0021] Mirmiran, P. , Fazeli, M. R. , Asghari, G. , Shafiee, A. , & Azizi, F. (2010). Effect of pomegranate seed oil on hyperlipidaemic subjects: A double‐blind placebo‐controlled clinical trial. British Journal of Nutrition, 104, 402–406. 20334708 10.1017/S0007114510000504

[vms31296-bib-0022] Ngo Njembe, M. T. , Dejonghe, L. , Verstraelen, E. , Mignolet, E. , Leclercq, M. , Dailly, H. , Gardin, C. , Buchet, M. , Waingeh Nain, C. , & Larondelle, Y. (2021). The egg yolk content in ω‐3 and conjugated fatty acids can be sustainably increased upon long‐term feeding of laying hens with a diet containing flaxseeds and pomegranate seed oil. Foods, 10, 1134. 34069647 10.3390/foods10051134 PMC8160806

[vms31296-bib-0023] O'fallon, J. V. , Busboom, J. R. , Nelson, M. L. , & Gaskins, C. T. (2007). A direct method for fatty acid methyl ester synthesis: Application to wet meat tissues, oils, and feedstuffs. Journal of Animal Science, 85, 1511–1521. 17296772 10.2527/jas.2006-491

[vms31296-bib-0024] Peebles, E. D. , & McDaniel, C. D. (2004). Practical manual for understanding the shell structure of broiler hatching eggs and measurements of their quality (Bulletin 1139). Mississippi Agricutural and Forestry Experiment Station.

[vms31296-bib-0025] Surai, P. F. , & Sparks, N. H. C. (2001). Designer eggs: From improvement of egg composition to functional food. Trends in Food Science & Technology, 12, 7–16.

[vms31296-bib-0026] Suzuki, R. , Noguchi, R. , Ota, T. , Abe, M. , Miyashita, K. , & Kawada, T. (2001). Cytotoxic effect of conjugated trienoic fatty acids on mouse tumor and human monocytic leukemia cells. Lipids, 36, 477–482. 11432460 10.1007/s11745-001-0746-0

[vms31296-bib-0027] Szymczyk, B. , & Szczurek, W. (2016). Effect of dietary pomegranate seed oil and linseed oil on broiler chickens performance and meat fatty acid profile. Journal of Animal and Feed Sciences, 25, 37–44.

[vms31296-bib-0028] Walzem, R. L. (1996). Lipoproteins and the laying hen: Form follows function. Poultry and Avian Biology Reviews, 7, 31–64.

